# Effect of invasive aspergillosis on risk for different causes of death in older patients with acute myeloid leukaemia or high-risk myelodysplastic syndrome

**DOI:** 10.1186/s12879-023-08013-5

**Published:** 2023-02-06

**Authors:** Rebecca van Grootveld, Valentina Masarotto, Peter A. von dem Borne, Nicole M. A. Blijlevens, Dana A. Chitu, Martha T. van der Beek, Marta Fiocco, Mark G. J. de Boer

**Affiliations:** 1 Department of Medical Microbiology, Leiden University Medical Center, Leiden, The Netherlands; 2 Mathematical Institute, Leiden University, Leiden, The Netherlands; 3 Department of Haematology, Leiden University Medical Center, Leiden, The Netherlands; 4 Department of Haematology, Radboud University Medical Center, Nijmegen, The Netherlands; 5 Department of Haematology, Erasmus University Medical Center, Rotterdam, The Netherlands; 6 Department of Biomedical Data Sciences, Leiden University Medical Center, Leiden, The Netherlands; 7 Department of Infectious Diseases, Leiden University Medical Center, Leiden, The Netherlands

**Keywords:** Aspergillosis, Invasive pulmonary aspergillosis, Invasive fungal infection, Acute myeloid leukaemia, Myelodysplastic syndromes, Aged

## Abstract

**Purpose:**

Study objectives were to estimate the cumulative incidence of death due to different causes of death (CODs) and investigate the effect of invasive aspergillosis (IA) on each separate COD in a cohort of older patients with acute myeloid leukaemia (AML) or high-risk myelodysplastic syndrome (MDS) included in the Haemato-Oncology Foundation for Adults in the Netherlands (HOVON) 43 randomized controlled trial.

**Methods:**

Pre-collected data from the trial was obtained from the HOVON data center and relevant clinical information was extracted. The cumulative incidence of death due to different CODs was estimated with a competing risk model and the association between each COD and prognostic factors, including IA, were investigated with a cause-specific hazard Cox regression model.

**Results:**

In total 806 patients were included, mean age of 70 years and 55% were male. The cumulative incidences of death due to leukaemia or infection at 3, 6, 12 and 36 months were 0.06, 0.11, 0.23, 0.42 and 0.17, 0.19, 0.22, 0.25 respectively. Incidence of IA was 21% and diagnosis of IA up until the final chemotherapy cycle was associated with an increased risk of dying from leukaemia (cause-specific hazard ratio (_CS_HR): 1.75, 95% CI 1.34–2.28) and a trend was seen for infection (_CS_HR: 1.36, 95% CI 0.96–1.91).

**Conclusion:**

Leukaemia was the most likely cause of death over time, however in the first year after diagnosis of AML or high-risk MDS infection was the most likely cause of death. Patients with IA had a relatively increased risk of dying from leukaemia or infection.

## Introduction

The incidence of invasive aspergillosis (IA) in patients with haematological malignancies ranges from 4 (with prophylaxis) to 11% (without prophylaxis) during remission induction chemotherapy and is approximately 8% after allogeneic stem cell transplantation (SCT) [1]. In patients with acute myeloid leukaemia (AML) the most common risk factor for development of IA is prolonged and severe neutropenia which is induced by AML or by intensive chemotherapy [2]. In addition, lack of response to chemotherapy and age have been reported to be a risk factors for development of IA [3–5].

AML is mostly seen in older patients with a median age of around 70 years [6, 7]. The 5-year survival for patients older than 15 years diagnosed with AML in Europe in 2000–2007 was 17% but survival rates decreased steeply with age [8]. Most patients with AML have active leukaemia at time of death. Other reported causes of death (CODs) include transplant related complications, infection (pneumonia in particular), bleeding, or other causes [9–14]. The contribution of IA to COD in patients with AML is difficult to quantify, because IA has multiple risk factors and these risk factors also directly contribute to AML treatment failure, complications and mortality [15, 16].

In the past years overall survival has improved for younger AML patients, mostly due to improved supportive care and improvements of allogeneic-SCT [17–19]. Conversely, for older patients with AML limited progress has been made [17–19]. This can be explained by patient and disease specific factors. Older patients with AML are more likely to have comorbidities, poor performance status, less tolerance to intensive chemotherapy and a higher incidence of poor prognostic factors, such as unfavourable cytogenetics and secondary leukaemia after myelodysplastic syndrome (MDS) [6, 20, 21]. Moreover, older patients are more susceptible for infections due to immunosenescence, a gradual decrease in immune system function associated with physiological aging that affects both the innate and the adaptive immune system, malnutrition and age related physiological changes [22, 23].

Study objectives were to estimate the cumulative incidence of death due to different CODs and to investigate the effect of IA on each separate COD in a cohort of older patients with AML (≥ 61 years) or high-risk MDS included in the Haemato-Oncology Foundation for Adults in the Netherlands (HOVON) 43 randomized controlled trial. The HOVON focuses on improving treatment methods for adult patients with malignant haematological disorders by implementing randomized controlled trials.

## Materials and methods

### Study design

Pre-collected data from the HOVON-43 randomized controlled trial [13] was requested from the HOVON data center. This trial included patients with AML or high-risk MDS. From all patients clinical information was extracted about diagnosis, treatment, response to treatment, survival and COD. The trial data contained an infection form in which all infections were registered during each chemotherapy treatment cycle. From the infection form, all patients with an occurrence of IA were identified. A competing risk model was applied to estimate the cumulative incidence of death with different competing events (different CODs). A cause specific hazard Cox regression model was used to investigate the association between survival time and multiple covariates.

All ethical considerations were within the intended use of the HOVON-43 randomized controlled trial and in accordance to the ethical principles as posed in the declaration of Helsinki as well as current national legislation.

### HOVON-43 trial data

Data was previously collected during the HOVON-43 randomized controlled trial that was performed from 2000–2006 [13]. This trial was performed to determine the optimal dose of daunorubicin in older patients (≥ 61 years old) treated for AML or high-risk MDS. In short, after diagnosis eligible patients were randomly assigned to receive a first cycle of conventional or escalated dose of daunorubicin in combination with cytarabine. Next, a second cycle of treatment was administered in both groups consisting of high dose cytarabine. After each cycle of remission induction chemotherapy response was assessed. Patients in complete remission after the second cycle could undergo a reduced intensity allogeneic-SCT if an HLA-matched donor had been identified. Otherwise, patients were randomly assigned to receive post remission chemotherapy with gemtuzumab ozogacimin (GO) or no treatment. Patients who were not in complete remission or who experienced a serious adverse event were not further randomized. Follow-up was performed until five years after the second chemotherapy cycle. Patients did not receive antifungal prophylaxis. At the time, chemoprophylaxis to prevent filamentous fungal infections was not standard practice.

### Patient population and data collection

A selection of baseline patient characteristics was made to investigate the effect of these prognostic factors on different CODs before start of chemotherapy. We obtained the following baseline variables: date of birth, gender, WHO performance status, AML or MDS, white blood cell (WBC) count at diagnosis, prior haematological or oncological disease, previous chemotherapy or radiotherapy, bone marrow cellularity, bone marrow fibrosis. Time dependent variables included outcome (death/alive), COD and occurrence of IA.

From the infection form the variable ‘occurrence of IA’ was created by identification of all patients with an occurrence of IA at any time point in the treatment schedule. Cases in which the diagnosis of IA was uncertain (n = 72) were discussed by two investigators (MdB and RvG) and final decision was based on consensus (17 IA; 55 no IA). In case consensus would not be reached, a third expert was consulted (MvdB). In most cases that were discussed only treatment with an antifungal agent was registered without mention of IA. That was not considered enough evidence for diagnosis IA. The uncertain cases that were classified as IA had a lung abnormality on imaging and were treated with an antifungal agent. The timepoint of IA was estimated from the chemotherapy cycle date in which the occurrence of IA was registered. Diagnosis of IA was made by the treating physician at the time. The data did not contain information about clinical characteristics, which diagnostic test was performed for the diagnosis of IA, or certainty of diagnosis according to EORTC/MSGERC guidelines [24].

In the dataset the COD was grouped into the following categories: leukaemia (including high-risk MDS), pneumonitis, infection, haemorrhage, veno-occlusive disease, secondary malignancy, other COD or unknown. Cause of death was recorded according to the clinical judgement of the treating physician at the time of death, as the rate of autopsy in this patient population was very low. In most patients the COD was described in a free text field as well. By checking the description of the COD, a few misclassified COD were reclassified.

For modelling purposes different CODs were grouped together. The variables ‘infection’ and ‘other COD’ were grouped together (‘infection and other COD’), because ‘other COD’ included 12/70 patients with infection as one of the documented CODs as well. In total, 158/216 (73%) in the group ‘infection and other COD’ had an infection. The variables ‘pneumonitis’, ‘haemorrhage’, ‘veno-occlusive disease’, ‘secondary malignancy’ and ‘unknown’ were grouped together as ‘miscellaneous’. In haematology patients pneumonitis is mostly defined as lung inflammation induced by chemotherapy. Therefore pneumonitis was not considered an infection.

### Statistical methods

In this study, a competing risk model [25] was applied to estimate the cumulative incidence of death with the competing events ‘leukaemia’, ‘infection and other COD’, and ‘miscellaneous’.

To study the association between each competing event (‘leukaemia’, ‘infection and other COD’ and ‘miscellaneous’) and prognostic factors a Cox proportional-hazard model [26] was estimated. Time fixed prognostic factors included in the model were AML or high-risk MDS, prior haematological or oncological disease, previous chemo- or radiotherapy, WBC count at diagnosis and age. Invasive aspergillosis was included in the model as time dependent. The last documented cycle of chemotherapy was selected for the diagnosis of IA. If a patient did not have IA from the time of inclusion until the end of the final chemotherapy treatment cycle, the patient was registered as not having IA. Hazard ratios (HR) along with their 95% confidence intervals (CI) were reported. A p-value of < 0.05 was considered statistically significant. Bone marrow cellularity and bone marrow fibrosis were not included as variable in the Cox proportional hazard model due to a high number of missing data, as was WHO performance status, because values were similar for all patients.

All analyses were performed in R software environment (https://www.R-project.org/) with the mstate library [27].

## Results

The HOVON-43 trial included a total of 866 patients. Overall, 60 patients were a priori excluded from the analysis (Table 1).

**Table 1:** Descriptive statistics of the selected relevant variables at time of diagnosis of AML or high-risk MDS

Characteristics	n (%)
Total	806
Age (mean)	70
Age	
≤70 years	427 (53)
>70 years	379 (47)
Sex	
Male	441 (55)
Female	365 (45)
AML or MDS	
AML	677 (84)
MDS	129 (16)
WHO performance status	
WHO 0	262 (33)
WHO 1	448 (56)
WHO 2	83 (10)
WHO 3	2 (0.2)
Bone marrow cellularity	
Low	60 (7)
Medium	123 (15)
High	430 (53)
WBC count (× 10^9 cells/L)	
1st quantile	2
Median	5.5
3rd quantile	27
Prior haematological or oncological disease	
No	747 (93)
Yes	59 (7)
Previous chemo- or radiotherapy	
No	596 (74)
Yes	210 (26)
Bone marrow fibrosis	
No	497 (62)
Yes	112 (14)

*AML* acute myeloid leukaemia, *MDS* myelodysplastic syndrome, *WBC* white blood cell. Unknown entry is not displayed

The HOVON trial included 866 patients of whom 60 were excluded from the analysis. Patients were excluded from the analysis for the following reasons: incomplete records (n = 33), time of diagnosis and or time of follow-up not documented correctly (n = 8), registered twice (n = 4), missing informed consent (n = 1), diagnosis of acute promyelocytic leukaemia (n = 6), no AML (n = 4), diagnosis of refractory anaemia with an international prognostic score of less than 1.5 (low-risk MDS) (n = 1), previously treated AML (n = 1), medical history of malignant lymphoma (n = 1) or concurrent liver cancer (n = 1)

Table 1 summarises the patient characteristics at baseline for the included 806 patients. The mean age at the time of diagnosis was 70 years (range: 61–88). In total, 710 (88.1%) patients died during the study period. Table 2 displays the number of patients and percentage for each COD. A total of 171 (21.2%) patients developed IA during follow-up, of whom 40 recovered. The remaining 131 patients had IA up until in their final chemotherapy cycle and of these patients 121 (92.4%) died. Most patients developed IA during remission induction chemotherapy in cycle I and II (Table 3). Table 2 shows the number and percentage of patients who had IA up until their final chemotherapy treatment cycle for the different CODs.

**Table 2:** Cause of death/alive grouped by patients with or without invasive aspergillosis up until their final chemotherapy cycle

Cause of death/Alive	With IA, n (%)	Without IA, n (%)	Total, n (%)
Total (known)	131 (100)	675 (100)	806 (100)
Leukaemia	59 (45)	321 (47.6)	380 (47.1)
Pneumonitis	10 (7.6)	22 (3.3)	32 (4)
Infection	31 (23.7)	115 (17)	146 (18.1)
Haemorrhage	5 (3.8)	30 (4.4)	35 (4.3)
Veno-occlusive disease	1 (0.8)	1 (0.1)	2 (0.2)
Secondary malignancy	1 (0.8)	9 (1.3)	10 (1.2)
Other	9 (6.9)	61 (9)	70 (8.7)
Unknown	5 (3.8)	30 (4.4)	35 (4.3)
Alive	10 (7.6)	86 (12.7)	96 (11.9)

The variables ‘infection’ and ‘other cause of death (COD)’ were grouped together (‘infection and other COD’), because ‘other COD’ included 12/70 patients with infection as one of the CODs as well. The variables ‘pneumonitis’, ‘haemorrhage’, ‘veno-occlusive disease’, ‘secondary malignancy’ and ‘unknown’ were group together as ‘miscellaneous’, because of the relatively small number of patients in all categories

**Table 3:** Chemotherapy treatment cycle in which invasive aspergillosis was first diagnosed

Chemotherapy treatment cycle	Number of patients that underwent chemotherapy treatment cycle, n	Cases of IA not recovered by the final chemotherapy cycle, n (%)	All IA cases, n (%)
Total	795	131 (16)	171 (22)
Cycle I	795	46 (6)	83 (10)
Cycle II	577	84 (15)	87 (15)
GO cycle I	114	0 (0)	0 (0)
GO cycle II	92	1 (1)	1 (1)
GO cycle III	65	0 (0)	0 (0)

*Cycle I and II* remission induction chemotherapy cycle I and II, *GO cycle I, II and III* gemtuzumab ozogacimin, post remission chemotherapy cycle I, II and III, *IA* invasive aspergillosis

Of note: Post-remission, 49 patients received reduced intensity allogeneic-stem cell transplantation (SCT) consolidation therapy. There were no data available about infections, or IA in particular, that occurred during or after SCT

**Fig. 1:**
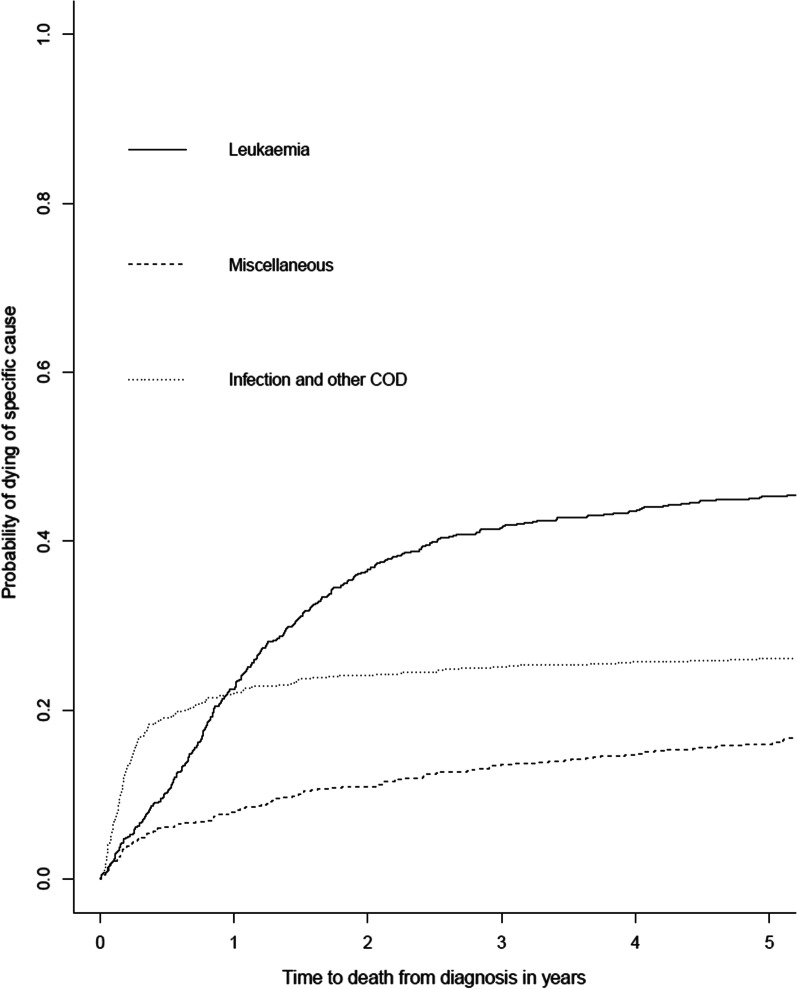
Cumulative incidence of different causes of death. The cumulative incidence of an event is displayed graphically as the plot of the cumulative incidence function. It displays the probability that a specific event (death due to leukaemia, infection and other cause of death (COD) or miscellaneous) has occurred by a specific time. Other COD included the CODs: leukaemia in combination with: infection (n = 8), infection and haemorrhage (n = 2), infection and multi organ failure (n = 1), haemorrhage (n = 2), pneumonitis (n = 2), cardiac disease (n = 2), respiratory insufficiency and pneumonitis (n = 1), respiratory and cardiac insufficiency (n = 1), pulmonary embolism (n = 1) or ileus (n = 1), and infection, renal failure and haemorrhage (n = 1), multiorgan failure (n = 8), liver failure (n = 1), renal failure (n = 1), cardiac disease (n = 14), respiratory insufficiency (n = 4), cardiac disease and haemorrhage (n = 1), acute respiratory distress syndrome (n = 2), graft versus host disease (n = 2), encephalopathy (n = 2), cerebrovascular accident (n = 3), pulmonary embolism (n = 2), ileitis (n = 1), hypoxic brain damage (n = 1), progressive supranuclear paralysis (n = 1), subdural bleeding (n = 1), progressive degeneration of general state (n = 1), euthanasia (n = 2) and suicide (n = 1). Miscellaneous included the CODs: pneumonitis, haemorrhage, veno-occlusive disease, secondary malignancy and unknown

Figure 1 displays the cumulative incidence of death for the different CODs. As expected from the summary presented in Table 2, leukaemia was the most likely COD over time. However, the probability of dying from an infection or other COD in the first year after diagnosis of AML or high-risk MDS was higher than the probability of dying from another cause.

The cause specific HRs (_CS_HR) estimated from the Cox model are provided in Table 4. Diagnosis of IA in the final cycle of treatment was associated with an increased risk of dying due to ‘leukaemia’ (_CS_HR 1.750 (95% CI 1.341–2.282)) and less markedly due to ‘infection and other COD’ (_CS_HR 1.359 (95% CI 0.958–1.909)). Age was included as a categorical variable in the model. Older age (> 70 years) seemed to have a protective effect for death due to leukaemia (_CS_HR 0.745 (95% CI 0.614–0.904)). Higher WBC count at diagnosis was significantly associated with an increased risk of dying from leukaemia. Note that WBC count was included as a linear term in the model, implying that the HR indicates the increase in risk for every unit of WBC count.

**Table 4:** Estimated cause-specific hazard ratio along with their 95% confidence interval for the different causes of death ‘leukaemia’, ‘infection and other cause of death’ and ‘miscellaneous’

Leukaemia	_CS_HR	95% CI	p-value
IA up until final chemotherapy cycle	1.750	1.341–2.282	< 0.001*
AML/MDS (MDS)	0.988	0.771–1.266	0.923
WBC count	1.002	1.001–1.004	0.054*
No prior haematological or oncological disease	0.938	0.751–1.169	0.567
No previous chemo- or radiotherapy	0.849	0.560–1.288	0.443
Age > 70	0.745	0.614–0.904	0.003*

Infection and other COD	_CS_HR	95% CI	p-value
IA up until final chemotherapy cycle	1.359	0.958–1.909	0.085
AML/MDS (MDS)	0.827	0.623–1.210	0.403
WBC count	1.002	0.996–1.005	0.106
No prior haematological or oncological disease	1.318	0.978–1.775	0.068
No previous chemo- or radiotherapy	1.320	0.870–2.003	0.191
Age > 70	0.961	0.757–1.210	0.747

Miscellaneous	_CS_HR	95% CI	p-value
IA up until final chemotherapy cycle	1.085	0.621–1.895	0.773
AML/MDS (MDS)	0.942	0.603–1.453	0.792
WBC count	0.999	0.997–1.005	0.733
No prior haematological or oncological disease	0.934	0.626–1.393	0.738
No previous chemo- or radiotherapy	0.538	0.231–1.258	0.153
Age > 70	0.855	0.607–1.206	0.373

*Statistically significant, *AML* acute myeloid leukaemia, *COD* cause of death, _*CS*_*HR* cause specific hazard ratio, *IA* invasive aspergillosis, *MDS* myelodysplastic syndrome, *WBC* white blood cell count, *95% CI* 95% confidence interval

## Discussion

### Cumulative incidence of death and effect of IA on death

We found that from 12 months onwards after diagnosis of AML or high-risk MDS leukaemia was the most likely cause of death. However, in the first 12 months after diagnosis of AML or high-risk MDS the probability of dying from an infection or other COD was higher than the probability of dying from another cause. By estimating the cumulative incidence with a competing risk model we were able to estimate the cumulative incidence for each COD and study the effect of prognostic factors on these CODs (’leukaemia’,’infection and other COD’, and’miscellaneous’). With regard to IA, patients with IA up until their final chemotherapy cycle had an increased risk of dying from ‘leukaemia’ or’infection and other COD’. This is in concordance with a study that reported that IA was associated with a higher risk of death in patients with AML as compared to patients without IA [10]. Another study showed that no development of IA during first remission induction chemotherapy was associated with complete remission and complete remission at three years [28].

The predisposing host risk factor for IA in patients with AML or MDS is prolonged and severe neutropenia [2, 29]. Neutropenia can be caused by intensive cytotoxic treatment or by the leukaemia itself. Patients with refractory leukaemia are at highest risk of developing IA, because they might be treated with multiple chemotherapy cycles and they might be neutropenic due to the underlying leukaemia [29]. These patients also have a higher risk of dying, because the presence of underlying disease, cytotoxic treatment and immunocompromised status can all can cause severe complications that lead to death. Invasive aspergillosis may often not be the direct COD. If death occurs in a patient suffering from multiple diseases, each disease can be either the direct COD (attributable), can have contributed to the events that lead to death (contributable), or be unrelated to death [30]. Despite, assessing the COD in patients with IA remains difficult and is further complicated because there is no universal approach to discriminate between association and causation with regard to IA and death [30].

### Incidence of invasive aspergillosis in older patients with AML or high-risk MDS

The incidence of IA in this cohort was 21% and IA occurred mostly during remission induction chemotherapy. This is similar to findings in other studies described in a recent meta-analysis, although incidences of IA ranged from 3.2%-43.1% during remission induction chemotherapy in patients without prophylaxis [1]. In this meta-analysis a crude incidence of 11.1% was reported during remission induction chemotherapy in patients without prophylaxis [1].

### Influence of age and other co-variables

The results from this study suggest that older age (> 70 years) had a slight protective effect with regard to the risk of dying due to leukaemia itself (_CS_HR: 0.745 (95% CI 0.641–0.904)). This was an unexpected finding since a higher incidence of poor prognostic factors is mostly found in older patients [6, 20, 21]. In an assessment of the outcomes of patients with secondary AML included in 13 EORTC trials conducted between 1986 and 2008, patients > 60 years did not have better outcomes than patients > 70 years. On the other hand, in this study WHO performance status was strongly associated with overall survival with a median of 0.8 years in patients with WHO performance status 0–1 and 0.4 years in patients with WHO performance status 2–4 [19]. It is unclear how these results should be explained. In our study, WHO performance status was comparable in patients ≤ 70 years and > 70 years. Thus, WHO performance status does not explain the slight protective effect of older age with regard to the risk of dying to leukaemia itself. The finding suggests that there might be a confounder, for example, differences in AML or MDS cytogenetics. Alternatively, the generalizability of the study might be limited by characteristics of the studied patient population. The finding that older age is protective may be biased by the phenomenon that less fit older patients may not have wanted to participate in the trial.

### Antifungal prophylaxis

At the time the HOVON-43 trial was performed antifungal prophylaxis was not prescribed to patients. Nowadays, *Aspergillus*-effective antifungal prophylaxis is standard of care in patients with AML or high-risk MDS undergoing intensive chemotherapy. Current national and international guidelines advise antifungal prophylaxis during prolonged neutropenia following chemotherapy for patients who are at high risk for IA [31–34]. In general, antifungal prophylaxis is not recommended beyond remission induction chemotherapy [32].

From the observed overall incidence of IA of 21% it becomes clear that antifungal prophylaxis would have been indicated in this group, in particular during remission induction chemotherapy. Only one patient developed IA during post remission chemotherapy. The reported incidence of IA during consolidation chemotherapy is low, around 2–3% [35, 36]. A retrospective multicentre study showed that antifungal prophylaxis during consolidation chemotherapy decreased the incidence of IA [35]. In this study, IA was diagnosed in 34/1137 (2.9%) who did not receive antifungal prophylaxis and in 22/1451 (1.5%) who did receive prophylaxis (p < 0.01) [35]. The number needed to treat to prevent IA was 71. The authors concluded that antifungal prophylaxis during consolidation chemotherapy may be required in older patients receiving high dose cytarabine, as age ≥ 60 years and treatment with high dose cytarabine were both independently associated with mortality. This only partly applied to our cohort. Although antifungal prophylaxis decreases the incidence of IA, IA cannot be fully prevented [1]. As an alternative to standard prophylaxis, screening strategies using a galactomannan serum assay can be deployed in high risk groups such as this study cohort [37].

### Study strengths and limitations

Strengths of the study included the size of the study population and the quality of the data which was prospectively collected during a randomized controlled trial. However, it should be considered that the cohort dates from 2006 and since then advances in diagnostics and treatment of IA as well as improvements in prophylactic strategies have been made. Still though, the competing risk analysis enabled us to study the effect of IA on mortality for each separate COD (’leukaemia’,’infection and other COD’ and’miscellaneous’). Furthermore, we focused on IA as COD in older patients with AML or high-risk MDS. Though there are some limitations. First, IA was described in the infection form, but in a small number of patients the diagnosis was uncertain. These cases were discussed and final decision was based on consensus. Secondly, no information was available about clinical characteristics, diagnostic tests that were performed, or the certainty of diagnosis. We were dependent on the diagnosis of IA that was made by the treating physician at the time and registered in the infection form. However, even though exact certainty of the diagnosis of IA could not be established in this cohort, as is more often the case in real-life clinical practice, the diagnosis (and treatment) of IA are usually not made and reported without consideration of host characteristics, imaging and clinical signs and symptoms. Thirdly, the COD was based on clinical diagnosis and not autopsy. In some cases, IA itself was the COD, but numbers are uncertain as COD infection was not always further specified. Fourthly, for modelling purposes different COD needed to be grouped together.

## Conclusions

In this cohort of older patients with AML or high-risk MDS, leukaemia was the most likely cause of death over time. However, in the first year after diagnosis of AML or high-risk MDS infection was the most likely cause of death. The high incidence of IA that was found underscores the indication for antifungal prophylaxis in this older patient population, in particular during remission induction chemotherapy. Since diagnosis of IA is not made and reported lightly in this population, the absence of the currently used EORTC classification system at the time, may only have had limited influence on the findings of this study. Patients with IA up until their final chemotherapy cycle had an increased risk of dying from leukaemia or infection, although the exact role IA played in the COD is unknown. Our results are suggestive of a contributable effect of IA, but further research is necessary that focusses on a universal classification to determine which role IA plays in the COD and the implementation of such a classification. Determining whether death is attributable, contributable or unrelated to IA will enhance our understanding of IA, and can lead to improved management of patients with leukaemia and IA.

## Data Availability

The data that support the findings of this study are available from the HOVON data center but restrictions apply to the availability of these data, which were used under license for the current study, and so are not publicly available. Data are however available from the authors upon reasonable request and with permission of the HOVON data center.
